# Illness Perception and the Severity of Depression and Anxiety Symptoms in Patients with Multimorbidity: Observational Cohort Studies

**DOI:** 10.3390/jcm13010069

**Published:** 2023-12-22

**Authors:** Sylwia Jaltuszewska, Gabriela Chojnacka-Szawlowska, Mikolaj Majkowicz, Sebastian Zdonczyk, Wojciech Homenda, Kazimiera Hebel

**Affiliations:** 1 Institute of Health Sciences, Pomeranian University in Slupsk, Westerplatte 64, 76-200 Slupsk, Poland; sylwia.jaltuszewska@upsl.edu.pl (S.J.); mikolaj.majkowicz@upsl.edu.pl (M.M.); wojciech.homenda@upsl.edu.pl (W.H.); 2 Faculty of Social and Humanities, WSB Merito University in Gdansk, Aleja Grunwaldzka 238A, 80-266 Gdansk, Poland; gchojnacka@wsb.gda.pl; 3 Institute of Pedagogy, Pomeranian University in Slupsk, Westerplatte 64, 76-200 Slupsk, Poland; sebastian.zdoncyk@upsl.edu.pl; 4 Provincial Specialist Hospital named after Janusz Korczak in Slupsk, Hubalczyków 1, 76-200 Slupsk, Poland

**Keywords:** perception, multimorbidity, mental health, chronic diseases, anxiety, depression

## Abstract

Introduction. Many studies have shown a correlation between the patient’s engagement in treatment and their perception of the illness. Aim: The aim of this study has been to explore the link between the patient’s perception of their illness with anxiety and depression, and to leverage this link to promote health education. Materials and methods: The study was carried out using the following tools: the Hospital Anxiety and Depression Scale and the Illness Perception Questionnaire–Revised. The study participants included N = 143 patients. Results: The participants’ age was statistically significantly associated with the expected duration of the illness (*p* < 0.01), the conviction that the treatment was effective (*p* < 0.01), and the perception of the severity of the disease symptoms (*p* < 0.05). The employment status was statistically significantly associated with the illness perception (*p* < 0.01). Anxiety and depression levels were statistically significantly associated with the perceived impact of the illness on life (*p* < 0.001) and emotional status (*p* < 0.001), the perceived control over the illness (*p* < 0.01), the potential for recovery (*p* < 0.001), the concern about the illness (*p* < 0.001), and the impact of the illness on emotional well-being (*p* < 0.001). Conclusions: Individuals who perceived a high severity of illness symptoms also assessed that the illness significantly impacted their life and emotional state. The authors demonstrate a strong link of a “negative” perception of the illness with depression and anxiety. A better understanding of the illness predicted a less severe depression and lower anxiety. Implications for practice: The results suggest that the study of illness perception holds significant potential to contribute effectively to educational and psychotherapeutic practices.

## 1. Introduction

Multimorbidity, also referred to as MCC—multiple chronic condition or coexisting conditions—is the presence of two or more chronic diseases in one person. Epidemiological data show that there is a growing number of people in modern society who suffer from simultaneous chronic conditions [1]. According to these authors, approx. 60% of adults meet the criteria of multimorbidity, while a report covering the last 30 years suggests that three out of five adults aged 65 and older suffer from three or more chronic diseases (with the median of five chronic diseases in one person). In 2013, multimorbidity affected 17.6% of Americans aged between 18 and 64 and 60.8% of Americans aged 65 and older [2]. Although multimorbidity statistics as reported by different authors may vary [3,4], the coexistence of multiple health conditions in one individual is one of the key challenges in healthcare. As such, multimorbidity is the focus of research that aims to, among other things, explore the cognitive, emotional, and behavioral processes related to the health condition of a person with multimorbidity or the quality of their life. It is believed that the knowledge in this area is still limited, mainly due to methodological challenges in identifying subjective and environmental variables, as well as their interactions with various coexisting disease processes and their impact on health [5]. The research carried out by McDaid et al. shows that the patterns of adaptation to coexisting chronic diseases are similar among patients from different counties (USA, Spain, and the UK) [5]. A study conducted on over 6000 patients in middle age with ischemic heart diseases, respiratory diseases, diabetes, rheumatic diseases, and chronic pain indicates that individuals with multiple health conditions were less physically fit, had a more negative perception of their health, and a lower quality of life, which is consistent with the results of other research in the field [5]. The impact of multiple health conditions on a patient’s perception of their health was, somewhat surprisingly, less significant than the impact of a single health condition. A study of over 3000 persons with coexisting health conditions aged between 65 and 85 shows that depression, somatization disorder, pain and limited activity, age, distress, and BMI index were inversely correlated with those persons’ perception of their health [6]. There was a stronger correlation of a high level of physical activity, high income, and the expectation of self-efficacy with the patient’s perception of their health than there was between the perception of health and the health conditions in this group of study participants [6]. This is why it was concluded that further research is needed to examine the correlation between multimorbidity and mental health more closely. In this context, it became crucial to create uniform standards required for the evaluation of the impact that multiple coexisting health conditions had on various areas of a person’s activity [7], and to examine the way in which the representation of the illness was created in the case of coexisting conditions [8]. The research plans were expanded to include the patients’ social communication, their life priorities, use of health care services, the quality of those services, and the expenses involved [7]. From an objective perspective, the implications of multimorbidity include factors such as a poorer quality of life, higher psychological distress, functional impairment, chronic pain, more frequent use of health care services, and a higher risk of premature death [4,9].

Although usually focused on the impact of a single disease on the various areas of the patient’s life, the research in this filed notes that the stress that accompanies multimorbidity requires different measures and actions to support the patient at different stages of the treatment process and the adjustment of the procedures applied by healthcare providers to the needs of patients with multimorbidity [7].

Efforts aimed at gaining a deeper understanding of the processes that individuals undergo when facing a health threat, during treatment, and after completing treatment are facilitated by the Common-Sense Model of Self-Regulation (CSM) developed by Leventhal et al. [10]. With increasing popularity in clinical practice, this model serves as the theoretical foundation that helps to explain an individual’s response when confronted with a health threat. The CSM model captures the dynamics of the multifaceted processes developed by individuals as a health threat representation, the mechanisms of coping with those threats, and proposes a plan of action and implementation [11,12,13,14]. The entire process is often initiated by somatic symptoms and deviations from normal functioning. The abovementioned stimuli trigger new or existing strategies, internalized patterns concerning expected functioning, as well as past experiences related to various illnesses and treatments. Different forms of activity that make up the lifestyle of an individual are also activated, contributing to the development of the so-called representation of a health threat, or, in other words, a mental image of this threat. The authors assigned these activities to several types of perceptions. They focus on the identification of the disease, its cause, control, outcomes, the perceived duration of the diseases, treatment options, and action plans related to the threat. Research results confirm that the individual representations of a health threat predict how a person will cope with the threat [15,16]. This model provides a foundation for the description and understanding of the processes at play when various activities are initiated and continued to contain the threat. It allows for the description of the dynamic interactions between the variables that control the health-related behavior of a person in response to an existing or future health threat. Based on this model, it is possible to predict the patient’s engagement in treatment and changes in their lifestyle to manage the health threat, whether assessed subjectively or objectively. Leventhal, Philips, and Burns [12] noted that this model’s utility was verified with respect to both the short-term symptoms of a health threat and potentially chronic health threats. However, there is no answer to the question of whether seeking medical help for experienced symptoms depends on the characteristics of the illness threat assessed in one or more dimensions of the model (for instance, the severity of symptoms and their cause, the sudden nature, the short or long duration, the level of the tendency for self-treatment, and the extent of the disruption of everyday life). Research results show that patients sought medical help early or decided not to seek medical help when they described the duration of the illness as short and the symptoms as mild and having a less-disturbing impact on everyday life. However, seeking medical help in connection with an analogous description of symptoms was also present in the study participants who described the health threat as chronic [12].

It appears that the CSM model offers a way to learn and predict the individual outcomes concerning the perception and adaptation by persons suffering from a single disease, as well as several coexisting health conditions. Research shows that an individual can perceive several diseases as a separate experience or as a set of experiences. Three models have been defined to describe the perception of coexisting health conditions. Model 1 covers the independent, main, and interaction effects of separate disease perceptions. In model 2, an individual prioritizes one disease over the others. The last model concerns individuals who merge the coexisting diseases into one general perception [17]. A study conducted on 2728 participants to verify the models showed that they were accurate to a similar extent, but each of them better explained a different set of variables. Model 1 explained the most variances in somatic activity. Perceived control had predictive value for somatic activity. The next model showed that individuals tended to merge several diseases into one multi-illness perception. The study findings regarding the structure of illness perception revealed the coexistence of disease-specific perceptions alongside a dominant influence of multiple diseases on perception. These perceptions were further shaped by personality traits, mood, and one’s disposition towards pessimism or optimism, as well as their varying levels of self-efficacy [18]. Given the challenges inherent in investigating the subjective and external factors influencing illness perception in individuals with multimorbidity, this study was undertaken to address the following questions:

1. Do sociodemographic variables influence illness perception in individuals with multimorbidity?
2. Does anxiety and/or depression influence illness perception in individuals with multimorbidity?
3. What is the structure of illness perception in individuals with multimorbidity based on Leventhal’s model?

In light of the aforementioned questions, it is worth noting that offering a subjective assessment of health falls within the purview of evaluations that enable us to track changes in a patient’s health over specific time periods, as well as gauge the influence of diseases and the effectiveness of treatment [19]. According to these researchers, the matter of disease perception is inherently complex, despite its seemingly straightforward nature, as it involves asking the patient about their present health condition and disruptions in both somatic and psychological activity. Illness perception constitutes the patient’s subjective evaluation, representing their unique perspective influenced by their expectations and aspirations. This implies that the changes reported by the patient do not necessarily correspond with the objectively assessed health condition, but could be attributed to shifts in their expectations and objectives. This process may be described as a “response shift” and it refers to the re-evaluation of the patient’s personal standards. It may obscure the positive or negative effects of treatment, for instance, due to the psychological adaptation to a chronic disease. As is well-known, the state of one’s health does not exist as a fixed point on an objective continuum that can be evaluated with precision. The perception of illness symptoms indicates that an awareness of the risks associated with chronic diseases and their consequences can frequently result in improved primary and secondary prevention as well as the treatment of such conditions [20,21,22,23].

However, the findings from the research in this field have, so far, exhibited inconsistency, and have yet to provide a comprehensive explanation to many issues. This holds particularly true for the understanding of the correlations of different chronic diseases with health-related practices, the perception of the illness threat, well-being, and the beliefs about the disease. It was discovered that sociodemographic variables significantly influenced the level of knowledge about diseases. However, the correlation between knowledge about diseases and health-related practices or health and well-being was found to be inconsistent or incomplete. 

The research that we have conducted here can contribute to the development of practical solutions that can be used in the treatment of patients with multimorbidity. It can also provide a practical insight into the potential for the effective application of the study of illness perception to educational and psychotherapeutic practices. Understanding the factors influencing illness perception will lead to the implementation of a treatment model that will rely on an individual approach to the patient. This could, in turn, improve the quality of life for these patients. 

## 2. Materials and Methods

This was a descriptive and observational cohort study. The cohort consisted of a sample of 143 patients selected from the population of patients hospitalized in chronic care units. The patients for the study were selected using the purposive sampling method, with participation in the study offered to each hospitalized patient who met the inclusion and exclusion criteria. The criteria for study participation included: being an adult, having expressed an informed consent for participation in the study, having received a primary disease diagnosis at least 6 months prior, and having at least one coexisting disease. The criteria for study disqualification included: the absence of coexisting diseases and a psychophysical condition that hindered participation in the study. The study was conducted at a time selected by each participant, and none of the participants withdrew from the study once it started.

The study was conducted according to the protocol approved by the bioethics committee.

The study was conducted using the following tools:

1. A conversation and a structured interview centered on gathering sociodemographic information, specifically focusing on age, gender, educational background, and employment status. The next question concerned the patient’s perceptions regarding the causes of their illness. The study participants provided their own answer to this question.
2. HADS (Hospital Anxiety and Depression Scale)—the original version authors: A.S. Zigmond, R.P. Snaith [24]. The Polish questionnaire’s reliability, as evaluated using the Cronbach alpha, ranged between 0.77 and 0.78 for the “Anxiety” scale, and 0.83 and 0.85 for the “Depression” scale, depending on the patient group. The reliability of the scales was assessed by examining the correlation between the emotional and social functioning scales of the EORTC QLQ-C30 questionnaire. The correlation coefficients were higher than 0.75 [25].
3. The illness perception was assessed using the Illness Perception Questionnaire. This study relied on the abridged version—the Brief Illness Perception Questionnaire. The individual items of this questionnaire represented subscales used to assess the perception of each of the model dimensions [26]. These authors report that, in the later findings of Leventhal’s and his colleagues’ research, it was noted that the illness perception is a whole dimension where the patient creates a multifaceted perception of their own illness. Illness perception was divided into several categories, with “identity” being characterized by the illness symptoms and name. The “timeline” dimension encompasses the illness’s gradual or sudden onset, its duration, variability, and fluctuations in the course of the illness. Perceptions about the causes of illness encompass factors such as genetics, infections, unhealthy diet, age, and other recognized contributors to illness. The “consequences” dimension of illness comprises a set of perceptions pertaining to the severity of the illness and its impact on various aspects of the patient’s life, including family, employment, and relationships. The “perceived control” dimension encompasses illnesses that were anticipated or those for which the patient believes they bear responsibility, as well as cases where the illness is attributed to the prescribed treatment. Another dimension, known as “illness coherence”, reflects the patient’s understanding of the illness. It has been observed that the representation of illness may exhibit varying degrees of incoherence depending on the specific illness prototype or prototypes involved. Another pertinent consideration is whether a patient constructs their illness representation based on the most up-to-date knowledge regarding diseases. The illness representation can undergo modifications as a consequence of interactions with healthcare professionals and interpersonal factors, including personality traits and individual differences.

### Data Analysis

Statistical analysis was performed using the STATISTICA Inc. (StatSoft Polska, Cracow, Poland (2022)), STATISTICA, version 13.3. The Shapiro–Wilk test was used to check whether the continuous variable was normally distributed (*p* > 0.05). The strength and direction of the relationship between two variables were determined based on Spearman’s correlation coefficient. One-way analysis of variance with post hoc NIR tests was used to determine whether people with different professional situations differed in their perception of their own illness. Factor analysis was used to determine the intensity of the experienced symptoms of the disease and the severity of depression and anxiety. The threshold for statistical significance for all tests was set at *p* = 0.05.

## 3. Results

### 3.1. Participant Characteristics

The study participants included N = 143 patients, with 80 patients (55.9%) receiving treatment in the hematology department, 50 patients (35%) receiving treatment in the nephrology department, and 13 patients (9.1%) receiving treatment in the internal medicine department. Out of the study participants, 59.4% were male, comprising 85 patients, while 40.6% were female, comprising 58 patients. The age of the study participants ranged between 19 and 92, with the age average at 53.8. The predominant educational background among participants was secondary education (54 patients, 37.7%) and vocational education (43 individuals, 30.1%). Individuals with higher education constituted a minority (32 patients, 22.4%). Professionally active individuals constituted 52.4% (75 patients), followed by 33.6% of retirees (48 persons). A small proportion included nonworking individuals (11 persons, 7.7%) and those on disability pensions (9 persons, 6.3%) (Table 1).

Among the study participants, 66% had cancer, with 58% of them having cancer as their primary disease. A total of 59.7% of the patients suffered from arterial hypertension, with 4.2% of the patients indicating it as the primary condition. Diabetes was observed in 30.6% of the patients, with 8.4% of them reporting it as the primary disease. Stage 4 chronic kidney disease was diagnosed in 46.6% of the participants, with it being the primary diagnosis for 19.6% of the individuals in the study group (Table 2).

The participants held diverse perceptions regarding the cause of the illness. As the cause of the illness, 24.5% of patients (35 people) mentioned lifestyle factors, followed by 16.1% (23 people) who cited genetic predisposition, 12.5% (18 people) mentioned coexisting conditions, and 11.2% (16 people) identified stress as the cause of the illness. Out of the entire group, 32 patients (22.4%) did not respond.

### 3.2. Results

Based on the results of Spearman’s rho correlation analysis, it can be concluded that the age of the participants was statistically significantly associated with the expected duration of the illness (r = 0.28; *p* < 0.01), the conviction that the treatment was effective (r = −0.27; *p* < 0.01), and the perception of the severity of the disease symptoms (r = 0.20; *p* < 0.05). Elderly individuals were more likely to believe that their illness would last for the rest of their lives and experienced more severe disease symptoms. Additionally, as the age increased, the belief that the disease could be overcome through treatment decreased.

Correlation analyses did not identify any statistically significant connections between patients’ education level and their perception of their illness. It was only found that, as the education level increased, the understanding of the illness also increased (r = 0.17; *p* < 0.05), but this correlation was not strong.

The next step was evaluating whether there were variations in the perception of illness among individuals with diverse employment statuses. A one-way analysis of variance with post hoc NIR tests was used for this purpose. Based on the results of these analyses, it can be concluded that employment status was statistically significantly associated with the perceived impact of the disease on life (F(3.139) = 4.76; *p* < 0.01), the perceived duration of the disease (F(3.139) = 4.89; *p* < 0.01), the conviction that the treatment could overcome the disease (F(3.139) = 7.40; *p* < 0.001), and the perceived severity of disease symptoms (F(3.139) = 4.83; *p* < 0.01). Those participants who were active in the job market perceived the least impact of the disease on their lives, had the least severe symptoms, and were more likely to believe that the disease could be overcome through treatment and would not last for the rest of their lives. No differences were found between individuals who were not working, on disability, or retired in terms of their perception of their illness. Therefore, the perception of one’s illness was associated with being professionally active.

Spearman’s rho correlation analysis was also used to examine whether there is a relationship between age and education with anxiety and depression. The patient’s age and education were not statistically significantly associated with the severity of their anxiety and depression. The only correlation demonstrated was that older individuals tended to experience a higher severity of anxiety r = 0.21; *p* < 0.05.

Using one-way analysis of variance, it was examined whether the employment status could affect the severity of anxiety and depression in the group of patients. The results of these analyses were statistically insignificant at *p* > 0.05, including in the case of the post hoc NIR tests. Therefore, it cannot be concluded that the severity of anxiety and depression varies among individuals in different occupational situations, including those who are employed, unemployed, retired, or receiving disability pensions. 

The study also aimed to ascertain whether there was a connection between patients’ perception of their illness and anxiety and depression. Based on the results of the analysis, it can be observed that the anxiety and depression scales were statistically significantly associated with the perceived impact of the illness on life (r = 0.38; *p* < 0.001), the perceived duration and chronicity of the illness (r = 0.29; *p* < 0.01), the perceived control over the illness (r = −0.23; *p* < 0.01), the potential for recovery (r = −0.36; *p* < 0.001), the perceived high severity of the symptoms (r = 0.39; *p* < 0.001), the concern about the illness (r = 0.39; *p* < 0.001), and the impact of the illness on emotional well-being (r = 0.60; *p* < 0.001). Individuals who perceived a high severity of illness symptoms also assessed that the illness significantly impacted their life and emotional state. Patients who believed that their illness could not be cured exhibited higher levels of anxiety and depression. 

The findings of this study also illustrate that anxiety was statistically significantly associated with the perceived impact of the illness on life (r = 0.35; *p* < 0.001), the chronicity of the illness (r = 0.30; *p* < 0.001), (negatively) with the perceived ability to control the illness (r = −0.24; *p* < 0.01), (negatively) with the perceived likelihood of curing the illness (r = −0.35; *p* < 0.001), (negatively) with the perceived high severity of the illness symptoms (r = 0.39; *p* < 0.001), the concern about the illness (r = 0.32; *p* < 0.001), and the impact of the illness on emotional well-being (r = 0.55; *p* < 0.001).

Similarly, the depression scale was also statistically significantly associated with the perceived impact of the illness on life (r = 0.35; *p* < 0.001), the perceived chronic character of the illness (r = 0.27; *p* < 0.01), and negatively with the perceived ability to control the illness (r = −0.31; *p* < 0.001), the perceived high severity of the illness symptoms (r = 0.33; *p* < 0.001), the concern about the illness (r = 0.43; *p* < 0.001), and the impact of the illness on the negative emotional state (r = 0.55; *p* < 0.001). 

The illness perception showed the most robust connection with the anxiety scale in the patients. The level of anxiety and depression in the examined patients was strongly associated with their belief in the impact of the illness on their emotional state, as well as with their perception of the symptom severity and concern about the illness (Table 3).

The factor analysis yielded two factors. With respect to factor 1, a significant relationship (with the factor loading level at >70) was found between the severity of the experienced illness symptoms and the intensity of depression and anxiety. The remaining variables related to the illness perception, albeit with lower factor loadings, suggest that factor 1 indicated strong associations of a “negative” illness perception with depression and anxiety. The factor loadings of factor 2 suggested that a better understanding of the illness was linked to a lower severity of depression (depressiveness) and anxiety (Table 4).

## 4. Discussion

This study illustrates that, during hospital treatment, in terms of experiencing illnesses with different pathomechanisms, clinical pictures, and treatment approaches, patients with multimorbidity generally exhibits an anxious and less-optimistic perception of their illnesses in reference to the dimensions of Leventhal’s model. It can be assumed that, during necessary hospital treatment, which objectively involves increased health risks, individuals who are removed from their usual way of life and separated from their loved ones perceive the stress of the illness with a significant presence of anxiety and depressive symptoms. However, whether we can attribute this to the social conditioning of anxiety and the relationship between anxiety and depressive symptoms would require separate studies [27]. 

The findings of this study illustrate that hospitalized patients with coexisting illnesses exhibited a varying structure of illness perception. Significant associations between the perceptions about the illness and the symptoms of anxiety and depression were found, aligning with the findings of other authors relating to individuals with multimorbidity [26]. Furthermore, it was observed that, the older the patient, the weaker the belief in the curability and controllability of the illness, and the stronger the belief in its extended duration. Older individuals also exhibited a more severe anxiety. A higher level of education was found to be associated with a stronger belief in understanding the illness. These relationships also applied to sociodemographic variables, as confirmed by the findings of other authors [20]. The association between older age and anxiety in the perception of illness as framed within Leventhal’s model may serve as an argument for exploring resources in the process of reducing or eliminating anxiety and depressive beliefs about the illness. For example, the results of this study indicate a positive perception of the illness among individuals who are employed.

An important finding was the absence of differences in the illness perception between women and men. This may provide valuable information for therapy, indicating that, as human beings, we either perceive the threat to life or not. Research conducted for many years has drawn attention to the biological and social context of differences in the evaluation of stress caused by health threat by women [28]. However, in-depth analyses are required to understand the representation of illnesses in individuals with depression, including women, among whom a negative mood impact on Leventhal’s model dimensions has been observed [29]. The findings of this study illustrate that anxiety and depression were associated with a more pessimistic set of beliefs in most dimensions of this model (these beliefs included, among others, the lack of influence on disease treatment, beliefs in its incurability, the destructive impact of the illness on daily activities, mood disturbances, and the presence of so-called negative emotions). Other authors have also noted such a perception of illness [29]. 

The results of this study, in relation to Leventhal’s model, have revealed that, as far as multimorbidity is concerned, it would be important to further understand the dynamics of changes in illness perception in the context of its clinical progression and changes in treatment approaches, especially in the context of the characteristics of coexisting diseases.

Individuals with coexisting illnesses experience multiple symptoms, which may be perceived as a greater burden, not simply the straightforward sum of the burdens associated with each individual disease. For example, pain and depression, as two common symptoms, can lead to a greater burden in individuals with multimorbidity. Pain in particular is considered the most common, intense symptom that elevates distress levels among such patients [9]. However, numerous studies published in the literature discussing cognitive illness representation focus on a single disease, unlike multimorbidity, which belongs to a less-explored aspect of this field. As our results, and those published by others, demonstrate, illness perception in the case of coexisting diseases poses a significant challenge for both patients and the healthcare professionals treating them. The interaction of symptoms from multiple diseases can also result in the failure of treatment. For example, depression, as mentioned earlier, is often comorbid in individuals with chronic illnesses, and can contribute to impaired functioning and poor adaptation to the illness. This can occur through pessimism, reduced sensitivity to illness symptoms, or excessive vigilance towards them, among others. On the other hand, effective treatment for depression among the mentioned group of patients can lead to improvements in various areas of their functioning [17].

The results of research on the structure of illness perception in individuals with coexisting diseases [8,24] reveal two variations: specific perceptions related to each disease and an overarching perception that is characteristic of the individual’s experience with multiple diseases, modified by subjective and contextual variables. Therefore, multimorbidity can be considered as the experience of the potential relationships (combinations) among diseases. In Leventhal’s common-sense model, it is believed that concepts related to illness representation are subjective. Therefore, it is important to understand the processes that lead different individuals to understand and interpret similar disease symptoms differently.

When it comes to this study’s results in the context of research by other authors, it can be stated that illness perception arises in relation to health threats, such as new somatic or psychological symptoms, a disease diagnosis, or a relatively new treatment approach. It involves emotional reactions and beliefs regarding this threat, such as the consequences, controllability, or the chronic nature of the disease. The results of the research by other authors highlight the importance of coping strategies and the influence of psychological and somatic factors on health outcomes [26]. The research results show variations in psychological and somatic symptoms relating to the same disease, and markers of disease severity do not always correlate with these differences [13,30]. Demonstrating the role and significance of anxiety, particularly depressive reactions, in increasing the motivation for treatment is a relatively novel idea in the relevant literature. Some items of illness perception according to Leventhal’s model are modifiable. Hence, a practical conclusion can be drawn regarding the potential effective application of the study of illness perception to educational and psychotherapeutic practices. The limitations of the study include a small patient group and the absence of a control group. For a deeper understanding of the topic, future research should involve an increased sample size, focus on specific medical diagnoses, and conduct studies in a longitudinal model. This would allow for an analysis of the dynamics in the perception of illness over time in the context of the progression of the disease and its consequences.

## 5. Conclusions

The illness perception of individuals treated in the hospital for coexisting diseases has revealed a wide range of beliefs about the illness. However, despite the pessimistic beliefs about the negative implications of their illness declared by some patients, they opted for hospital treatment. 

The findings of this study illustrate that anxiety and depression were associated with a more pessimistic set of beliefs in most dimensions of Leventhal’s model (these beliefs included, among others, the lack of influence on disease treatment, beliefs in its incurability, the destructive impact of the illness on daily activities, mood disturbances, and the presence of so-called negative emotions).

The results obtained indicate that, the older the patient, the weaker the belief in the curability and controllability of the illness, and the stronger the belief in its extended duration.

A higher level of education was found to be associated with a stronger belief in understanding the illness.

The results obtained indicate the absence of differences in illness perception between women and men. This may provide valuable information for therapy, indicating that, as human beings, we either perceive the threat to life or not.

## Figures and Tables

**Table 1:** Participant characteristics.

Variables		N	%
Sex	Female	58	40.6
	Male	85	59.4
Age	30 or younger	19	13.3
	31–40 years old	21	14.7
	41–50 years old	20	14.0
	51–60 years old	21	14.7
	61–70 years old	34	23.7
	71–80 years old	23	16.1
	80 or older	5	3.5
Education	Primary education	14	9.8
	Vocational education	43	30.1
	Secondary education	54	37.7
	Higher education	32	22.4
Employment	Unemployed	11	7.7
	Employed	75	52.4
	Disability pension	9	6.3
	Retired	48	33.6

**Table 2:** The percentage of diagnosed conditions among individuals in the study group.

	Cancer	CKD * (Stadium 4)	Diabetes	Arterial Hypertension	Ischemic Heart Disease	Gout	COPD *	Liver Cirrhosis	Rheumatoid Arthritis	Thyroid Diseases	Other Digestive System Diseases	Other Respiratory Diseases	Other Diseases
Primary diagnosis	58.0	19.6	8.4	4.2	3.5	0.7	0.7	0.7	0.7	0.0	10.4	0.0	2.1
Coexisting condition	8.0	27.0	22.2	55.5	9.5	4.8	4.8	7.9	6.3	6.3	9.5	4.8	30.2
In total	66.0	46.6	30.6	59.7	13.0	5.5	5.5	8.6	7.0	6.3	19.9	4.8	32.3

* CKD—chronic kidney disease; COPD—chronic obstructive lung disease.

**Table 3:** The results of the correlation analyses for the relationship between illness perception and anxiety and depression.

	The Hospital Anxiety and Depression Scale	Anxiety	Depression
To what extent does the illness you are suffering from affect your life?	0.38 ***	0.35 ***	0.35 ***
How long do you think your illness will last?	0.29 **	0.30 ***	0.27 **
To what extent are you able to control your illness?	−0.23 **	−0.24 **	−0.16
How much do you think treatment can help you overcome your illness?	−0.36 ***	−0.35 ***	−0.31 ***
How severe do you feel your symptoms are?	0.39 ***	0.39 ***	0.33 ***
To what extent are you worried about your illness?	0.39 ***	0.32 ***	0.43 ***
How much do you think you understand your illness?	−0.06	−0.11	−0.01
To what extent does the illness affect you emotional well-being?	0.60 ***	0.55 ***	0.55 ***

** *p* < 0.01; *** *p* < 0.001.

**Table 4:** Factor analysis.

Variable	Factor 1	Factor 2
To what extent does the illness you are suffering from affect your life?	0.65	0.11
How long do you think your illness will last?	0.49	0.40
To what extent are you able to control your illness?	−0.14	0.77
How much do you think treatment can help you overcome your illness?	−0.57	0.10
How severe do you feel your symptoms are?	0.70	0.29
To what extent are you worried about your illness?	0.66	0.25
How much do you think you understand your illness?	0.08	0.75
How much does your illness affect your emotional well-being?	0.79	0.04
HADS—anxiety	0.77	−0.33
HADS—depression	0.76	−0.21

Explanation: method: principal components; minimum eigenvalue: 1.000; factor rotation: Varimax normalized; factor loadings for analysis > 7.

## Data Availability

Data can be obtained from kazimiera.hebel@upsl.edu.pl.
